# Chloroquine/Hydroxychloroquine Use and Suicide Risk: Hypotheses for Confluent Etiopathogenetic Mechanisms?

**DOI:** 10.3390/bs11110154

**Published:** 2021-11-07

**Authors:** Alessandra Costanza, Valeria Placenti, Andrea Amerio, Andrea Aguglia, Gianluca Serafini, Mario Amore, Elena Macchiarulo, Francesco Branca, Roberto Merli, Guido Bondolfi, Khoa Dinh Nguyen

**Affiliations:** 1 Department of Psychiatry, Faculty of Medicine, University of Geneva (UNIGE), 1211 Geneva, Switzerland; Guido.Bondolfi@hcuge.ch; 2 Department of Neuroscience, Rehabilitation, Ophthalmology, Genetics, Maternal and Child Health (DINOGMI), Section of Psychiatry, University of Genoa, 16132 Genoa, Italy; valeria.placenti91@gmail.com (V.P.); andrea.amerio@unige.it (A.A.); andrea.aguglia@unige.it (A.A.); gianluca.serafini@unige.it (G.S.); mario.amore@unige.it (M.A.); 3 IRCCS Ospedale Policlinico San Martino, 16132 Genoa, Italy; 4 Department of Mental Health, Mental Health and Suicide Prevention Center, 13900 Biella, Italy; elena.macchiarulo@aslbi.piemonte.it (E.M.); francesco.branca@aslbi.piemonte.it (F.B.); roberto.merli@aslbi.piemonte.it (R.M.); 5 Department of Psychiatry, Service of Liaison Psychiatry and Crisis Intervention (SPLIC), Geneva University Hospitals (HUG), 1211 Geneva, Switzerland; 6 Hong Kong University of Science and Technology, Hong Kong, China; khoa.d.nguyen@gmail.com; 7 Tranquis Therapeutics, Palo Alto, CA 94304, USA; 8 Department of Microbiology and Immunology, Stanford University, Palo Alto, CA 94304, USA

**Keywords:** chloroquine, hydroxychloroquine, suicide, suicidal behavior, suicidal ideation, suicide attempt, COVID-19

## Abstract

Chloroquine (CQ) and hydroxychloroquine (HCQ) are classical anti-malarial and anti-inflammatory treatments, which were used as first-line therapy at the beginning of the 2019 coronavirus disease (COVID-19) pandemic. Besides the emerging data on their lack of efficacy against COVID-19 infection, such treatments have been associated with some severe health concerns, including those of neuropsychiatric nature, such as a possible increase in suicide risk. Here we report a case of a patient with no history of psychiatric illnesses, who abruptly developed depression with melancholic features, severe suicidal ideation (SI), and attempted suicide (SA) shortly after receiving HCQ for his COVID-19 infection. The case was followed by a mini-review of the heterogeneous scientific literature on the hypothetical association between neuropsychiatric symptoms, with a focus on SI and suicidal behavior (SB, including SA and death by suicide), when CQ and HCQ are used in COVID-19, rheumatologic diseases, and malaria settings. Considering the anti-inflammatory properties of CQ and HCQ and the implications for neuroinflammation in suicide pathogenesis, the possible increase in suicide risk caused by these medications appears paradoxical and suggests that other underlying pathological trajectories might account for this eventuality. In this regard, some of these latter mechanistic postulates were proposed. Certainly the role and contribution of psycho-social factors that a COVID-19 patient had to face can neither be minimized nor excluded in the attempt to understand his suffering until the development of SI/SB. However, while this case report represents a rare scenario in clinical practice and no consensus exists in the literature on this topic, a psychiatric screening for suicide risk in patients using of CQ and HCQ could be carefully considered.

## 1. Introduction

Chloroquine (CQ) and hydroxychloroquine (HCQ) are 4-aminoquinoline-derived agents, initially developed as antimalarial drugs. Due to their anti-inflammatory properties, both HCQ and CQ are also currently used for the treatment of chronic inflammatory and autoimmune diseases such as rheumatoid arthritis and systemic lupus erythematosus [1]. These pharmacological agents are known to be responsible for a wide range of side effects, including retinopathies, myopathies, and cardiotoxicity [2,3,4]. Although infrequent, neuropsychiatric adverse events (AE) have also been reported in patients using CQ and HCQ since the early 1960s [5]. These AEs were first observed in travelers taking CQ or HCQ as antimalarials as well as in subjects without history of psychiatric illnesses. However, the role of premorbid vulnerability remains unclear in these cases.

Following the 2019 coronavirus disease (COVID-19) outbreak, medical researchers began investigating the role of CQ or HCQ as possible treatment for COVID-19. Given their in vitro antiviral activity against a clinical isolates of 2019-nCoV [6], both drugs were approved as emergency treatment for COVID-19 hospitalized patients by the Food and Drug Administration (FDA) and the European Medicines Agency (EMA) at the beginning of the pandemic [7,8]. However, in June 2020, based on the emerging scientific data which highlights an increased risk of serious AEs and a substantial lack of clinical efficacy in treating COVID-19 [9,10], the FDA revoked the emergency use authorization (EUA) of CQ or HCQ as treatment for hospitalized COVID-19 patients outside of clinical trials [11]. Furthermore, with the widespread use of CQ and HCQ for the treatment of COVID-19 infections in many countries, concerns regarding their risk of neuropsychiatric AEs have been raised.

In light of this emerging health concern in the context of the COVID-19 pandemic, we described a case report and a mini-review on the current literature on psychiatric AEs signalized to the use of CQ and HCQ, with a particular focus on possible suicide risk (suicidal ideation (SI) and behavior (SB), including suicide attempts (SA) and death by suicide) and their putative etiopathogenic mechanisms related to these drugs.

## 2. Case Report

A 54-year-old man was diagnosed with SARS-CoV2 infection during the first wave of the pandemic. The patient had a mildly elevated temperature (37.5 °C which did not require administration of paracetamol), reported an incomplete loss of taste and smell, and had no cardiac/pulmonary involvement. The infection did not result in any serious complications that required hospitalization and HCQ was prescribed (200 mg twice a day) by his general practitioner. The patient had no personal or family history of psychiatric illnesses as well as other somatic comorbidities, except for his diabetes mellitus type 2, which was treated with metformin. The patient had a supportive family and social network. He was neither directly affected by the sanitary emergency (such as bereavement) nor presented with any financial stressors (such as fear of job loss or status decline). He also did not report any concerns about his COVID-19 prognosis. After five days of HCQ treatment, the patient suddenly experienced depressive symptoms with melancholic features and severe SI. Subsequently, the patient attempted jumping off his balcony when he thought he was alone, but his SA was interrupted by his son.

After his SA, the patient was hospitalized in a COVID-19 unit with constant surveillance and psychiatric consultations. The patient started receiving psychopharmacological treatment with sertraline 50 mg/day and olanzapine 5 mg/day. HCQ treatment was stopped. The initial dosing regimen of sertraline was scheduled to be increased in the following days. However, around one week after the discontinuation of HCQ, his psychiatric conditions progressively and significantly improved. Particularly, his severe depression subsided, and SI completely disappeared. Therefore, sertraline was not augmented as planned and olanzapine was reduced to 2.5 mg/day for one week and eventually withdrawn.

In retrospect, the patient described the events (including deep depressive suffering accompanied by SI and interrupted SA) that led him to the psychiatric hospitalization as a “black-out” or a “nightmare” that began immediately after the initiation of HCQ treatment. He was discharged from the psychiatric unit and continued his psychiatric follow-up visits in an outpatient mental health service. His psychic conditions remained stable in the following months. After 1 year, sertraline was discontinued, without any reoccurrence of psychiatric symptoms.

## 3. Discussion

Here we report the case of a patient with no history of psychiatric illnesses, who developed a sudden major depressive episode with melancholic features, severe SI and SA, shortly after receiving HCQ for his COVID-19 infection. The rapid improvement in these neuropsychiatric symptoms coincided with the interruption of HCQ treatment. Sertraline was also introduced; however, the clinical efficacy of selective serotonin reuptake inhibitors (SSRIs) could only be detected after about 3 weeks of treatment. The severity of his major depressive episode with melancholic features and complete imperviousness to environmental positive inputs, suggested an important biological contribution to its origin. Furthermore, his psychiatric condition might not have an important psychosocial origin, as the patient experienced no stressors of socioeconomic or psychological nature and did not belong to more vulnerable populations [12,13,14], as those who were widely described in literature [15,16]. We considered the following differential diagnoses. (i) Major depression with melancholic characteristics, first depressive episode: this was discarded due to the sudden onset after introduction of HCQ and the unusually rapid resolution after cessation of HCQ; (ii) first major depressive episode in the context of a relatively late-onset bipolar disorder: this was discarded based on the same reasoning given in (i); moreover the longitudinal follow-up did not reveal any further hypomanic, manic, or depressive episode; (iii) onset of a cerebral lesion (e.g., brain tumor) or a neuro-degenerative disease (e.g., fronto-temporal dementia that is associated to SI and SB): they were excluded following neuroimaging and a psychometric evaluation; (iv) impact of psycho-sociological factors: this did not apply to our patient, whose relatives, closest friends, and family doctor stated that he was unconcerned by the prognosis, and also the patient himself reported in a sincere and authentic manner that he had no concerns about the prognosis, especially since it was immediately shown to be favorable; furthermore, the patient described himself as lucky from a somatic point of view compared to other patients with adverse prognoses; (v) impact of concerns, particularly the fear of death: this was discarded based on the same reasoning given in (iv). While it is not possible to rule out the impact of COVID-19 lockdown measures as well as possible underlying fear of the long-term consequences of COVID-19 infection on the development of his psychiatric complications, considering the reciprocal interactions between biological and non-biological factors [17,18,19,20], non-biological factors seem largely insufficient to explain the chronology and severity of the presented depressive and suicidal symptomatology. Collectively, an HCQ AE at the basis of both severe depression and SI/SB could be hypothesized.

CQ/HCQ have been associated with several neuropsychiatric AEs in rheumatic diseases, infectious diseases, and COVID-19 [21,22,23,24,25,26,27,28,29]. These AEs can range from relatively mild psychiatric symptoms, such as irritability, nervousness, and insomnia, to intermediate and severe clinical conditions including adjustment disorder, anxiety also with pervasive features, “personality change”, major depression, psychosis, confusion, psycho-motor agitation, delirium, and neurocognitive disorders [21,22,23,24,25,26,27,28,29] (Table 1). Notably, some evidence of increased risk of SI/SB has been described [21,22,27,28]. This risk was particularly signalized among patients in co-treatment with metformin or elderly (possibly due to diminished neuronal reserve and presence of mixed neurodegenerative and vascular brain injuries) [23,26,30]. However, these findings have not been corroborated in other studies [24,25,28]. Of interest to its neuropsychiatric AEs, CQ/HCQ exhibits neurotropism as CQ level in the CNS was shown to be 10–30 times higher than its serum concentration after dosing [31]. This phenomenon might be attributed to the interaction of CQ with the P-glycoprotein at the blood–brain barrier interface [32]. Since CQ and HCQ exhibit similar physiochemical properties, many pharmacokinetic features of HCQ are often inferred from CQ studies [1].. Given the neurotropic impact of CQ/HCQ, all studies recommend informing patients and their relatives about this possible increase in suicide risk without discouraging the necessary use of CQ/HCQ for the underlying pathological conditions [21,22,23,24,25,26,27,28,29].

**Figure 1:**
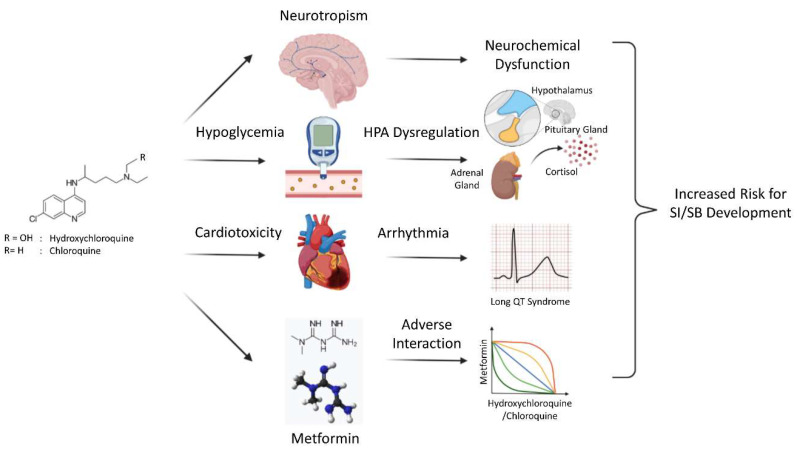
Putative mechanisms of HCQ/CQ-induced neuropsychiatric adverse events. Biochemical properties of HCQ/CQ can cause several side effects that might contribute to the development of SI/SB. Its accumulation in the brain can cause disruption of neurotransmitter signaling circuits involved in the pathogenesis of suicide. Its metabolic and cardiovascular impacts can result in abnormal cortisol release and arrythmia, both of which have been linked to increased suicide risk. The adverse interaction of HCQ/CQ with metformin might also precipitate in fatal neuropsychiatric outcomes. Legend: SI = suicidal ideation; SB = suicidal behavior; HPA = hypothalamic-pituitary-adrenal axis.

Since markers of inflammation and dyslipidemia, such as CRP, IL-1, IL-6, TNF-a, and cholesterol levels, have been suggested as etiological risk factors of SI/SB development [33,34,35,36], the possible association of neuropsychiatric complications with CQ/HCQ, which exert potent anti-inflammatory [1] /anti-lipidemic properties [37,38] appears paradoxical and suggests that other underlying pathological mechanisms might account for these phenomena (Figure 1). In this regard, CQ and HCQ have been proposed to (i) induce neurochemical interference with calcium signaling in neural cells [39] as well as disruption of dopamine and acetylcholine homeostasis [40,41], and ultimately precipitating into neuropsychiatric symptoms, including suicide (Figure 1). Alternatively, several other well-documented side effects of HCQ might also potentiate the risk of developing neuropsychiatric AEs, particularly SI/SB. For instance, (ii) HCQ treatment can cause hypoglycemia [42], which could trigger dysregulation of the hypothalamic-pituitary-adrenal (HPA) axis, and, subsequently, cortisol rhythm [43,44] (Figure 1). Notably, alterations in these signaling pathways have been associated with increased suicide risk [45,46,47]. (iii) Another side effect of HCQ is its cardiotoxicity, which has been mainly attributed to its impact on arrhythmia (QT interval prolongation) [4,48,49] (Figure 1). In this regard, heart diseases and abnormal heart rate variability have been suspected to be a risk factor for SI/SB development. Specifically, elevated suicide rates have been found in subjects with cardiac comorbidities of heart failure, cardiomyopathy, acute myocardial infarction, cardiac arrest, angina pectoris, tachycardia, and atrial fibrillation and flutter [50]. In addition, root mean square of successive difference, a measurement of temporal variations in normal heart rhythms, and resting heart rate have been associated with moderate-to-high suicide risk, based on the mini-international neuropsychiatric interview (MINI) suicide scores [51]. Therefore, it is plausible to postulate that HCQ-associated cardiotoxicity might augment the risk of suicide in patients receiving this medication. (iv) Last but not least, a novel drug-drug interaction has been identified between HCQ and metformin [23] (Figure 1). In a study of 10,771 case reports of safety concerns related to HCQ, metformin and HCQ coadministration was shown to result in fatal outcomes, particularly death by suicide. This observation is of particular relevance to the present case as the patient had been treated for his diabetes with metformin and therefore would be at a high risk for fatal outcomes, such as SI/SB. This adverse interaction has been hypothesized to be the result of autophagic inhibition by metformin/chloroquine as an increase in autophagosomes in hearts, livers, and kidneys of animals treated with these two medications was linked to their fatal toxicity [52]. Additionally, the synergy between metformin and HCQ might potentiate the risk of developing severe hypoglycemia, causing heightened HPA dysregulation and, ultimately, increasing suicide risk.

An important limitation of this case report is the consideration that a pandemic condition in itself may lead to SI and SB via several mostly psycho-social pathways, such as anxiety, depression, post-traumatic stress disorder (PTSD), concerns (e.g., fear/worry of contracting the virus), pessimism, worthlessness, sleep problems, financial strain/insecurity, domestic violence, helplessness, isolation, loneliness, and disconnectedness [19,53]. In this context, we did not explicitly consider fear of death as a concern caused by the pandemic in the described patient. However, this was not an omission but a result of our assessment of the patient (see item (iv) in the differential diagnosis paragraph). While the important role of fear of death still remains relatively less analyzed and empathized among patients in analogy to what also occurs in other settings where the integrity of the person is threatened, such as emergency departments [54,55], more studies have investigated this concept in healthcare workers, typically in relation with hopelessness and PTSD [14,56]. We postulate that in the patient described, psychosocial stressors played a less relevant role than the chronological concomitance of symptomatology with HCQ intake as these factors appear to be extremely favorable from a psychological, social, and prognostic point of view. Nevertheless, the onset of psycho-social triggers and fear of death, when faced with an unexpected, unfamiliar condition with possible tragic consequences—even if unexpressed—cannot be excluded categorically in having contributed to the patient’s SI and SA.

## 4. Conclusions

Our case report highlights some infrequent, yet serious neuropsychiatric complications, including SI/SB, of HCQ in a COVID-19 patient. Certainly the role and contribution of non-biological factors that a COVID-19 patient had to face can neither be minimized nor excluded in the attempt to understand his suffering until the development of SI/SB. However, a psychiatric screening for suicide risk in patients using of CQ and HCQ could be carefully considered. While various biological factors related to the “off-target” effects of HCQ might be accountable for these AEs, further data are needed to provide unequivocal mechanistic insights into the risks associated with this controversial COVID-19 therapy.

## Figures and Tables

**Table 1:** Neuropsychiatric manifestations and SI/SB related to CQ/HCQ treatment (studies specifically investigating the possible association with SI/SB were reported).

Authors	Psychiatric AEs	SI/SB	Use
Garcia et al., 2020 [21] 1754 reports of HCQ treatment in COVID-19(VigiBase ICSRs *)	56 cases (50% considered serious: 12 cases of psychosis and 7 cases of SB, see beside); others: insomnia, anxiety, and confusion. Increased risk of psychiatric AEs with HCQ vs. other antiviral drugs (ROR 6.27, CI 2.74–14.35, 95%)	4 deaths by suicide (men, within 4 days after HCQ treatment) 3 cases of suicidal self-harm	C
Hamm et al., 2020 [22] Literature review of HCQ treatment in COVID-19 (PubMed 1950–2020)	Psychosis (most studied); delirium, adjustment disorder (most common); anxiety (weak association)	Weak association with increased suicide risk Suicide risk should be screened prior to CQ/HCQ prescription	C
Montastruc et al., 2020 [23] Disproportionality analysis of 10,771 reports of CQ/HCQ treatment with metformin and other antidiabetic medications(VigiBase ICSRs *)	-	HCQ-metformin interaction associated with fatal outcomes, particularly death by suicide (in comparison with HCQ alone, HCQ + metformin: ROR 57.7, CI 23.9–139.3, 95%; in comparison with metformin alone, HCQ + metformin: ROR 6.0, CI 2.6–13.8, 95%)	C
Sato et al., 2020 [24] Disproportionality analysis of 2,389,474 cases with CQ/HCQ treatment (FAERS database)	- 520 cases Significant higher reports of delirium, loss of consciousness, amnesia, hallucinations, and depression (ROR > 1, 95%) No statistically significant higher reporting of other neuropsychiatric AE, including psychosis and agitation	No association with increased suicide risk	C
Lane et al., 2021 [25] Cohort studies of 918,144 cases with HCQ vs. 290,383 cases with other medications (records from 10 sources in Germany, UK, and USA)	No association with depression and psychosis with both short and long-term use	No association with increased SI/SB with both short and long-term use	R
Mascolo et al., 2018 [26] Literature review of HCQ treatment in elderly patients	Psychiatric AEs depend on different risk factors in patients with autoimmunity	Patients and their relatives should be informed of the risk of developing SB and other neuropsychiatric AEs and eventually of the possibility of switching medications However, these possible AEs should not discourage the use of HCQ in a rheumatological setting	R
De Oliveira Ribeiro et al., 2014 [27] Naturalistic study of rheumatoid arthritis patients treated with HCQ and other medications	Increased risk of anxiety and depression with HCQ or biological drugs vs. leflunomide/methotrexate (*p* < 0.001)	Increased SI risk with HCQ or biological drugs vs. leflunomide /methotrexate (*p* < 0.001)	R
Meier et al., 2004 [28] Observational study of 35,370 patients with CQ/HCQ and other medications (medical records)	Low risk of psychosis and panic attacks with all antimalarial medications No association of mefloquine with depression when compared with other antimalarial medications	2 deaths by suicide (men, treated with mefloquine)	M
Good et al., 1977 [29] Literature review of patients with CQ/HCQ treatment	Psychosis, “personality change”, depression and delirium	SI could be consequence of CQ treatment	M

Legend: AE = adverse events; SI/SB = suicidal ideation/suicidal behavior; C = COVID-19; R = rheumatologic disorders; M = malaria; CQ = chloroquine; HCQ = hydroxychloroquine; ROR = reporting odds ratio; CI = confidence interval; ICSRS = individual case safe reports included in the World Health Organization’s pharmacological database; FAERS = Food and Drug Administration Adverse Event Reporting System. * VigiBase Study = the World Health Organization’s pharmacological database including ICRSs all from more than 130 countries around the world.
